# Targeting the renin-angiotensin system to improve cancer treatment: Implications for immunotherapy

**DOI:** 10.1126/scitranslmed.aan5616

**Published:** 2017-10-04

**Authors:** Matthias Pinter, Rakesh K. Jain

**Affiliations:** 1 Edwin L. Steele Laboratories for Tumor Biology, Department of Radiation Oncology, Harvard Medical School and Massachusetts General Hospital, Boston, MA 02114, USA; 2 Division of Gastroenterology and Hepatology, Department of Internal Medicine III, Medical University of Vienna, Vienna, A-1090, Austria

## Abstract

Renin-angiotensin system (RAS) inhibitors (RASi)—widely prescribed for the treatment of cardiovascular diseases—have considerable potential in oncology. The RAS plays a crucial role in cancer biology and affects tumor growth and dissemination directly and indirectly by remodeling the tumor microenvironment. We review clinical data on the benefit of RASi in primary and metastatic tumors and propose that, by activating immunostimulatory pathways, these inhibitors can enhance immunotherapy of cancer.

## INTRODUCTION

**Fig. 1:**
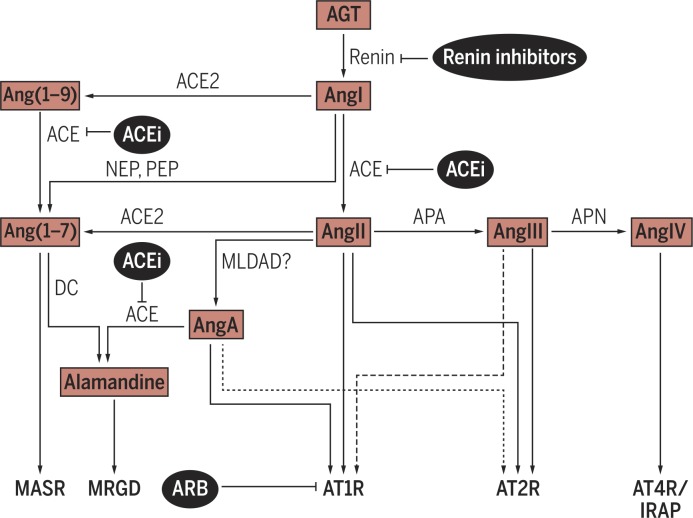
**The RAS is a complex system whose bioactive peptides signal through different receptors**. Angiotensinogen (AGT), generated and released into circulation by the liver, is hydrolyzed by renin, a product of the kidneys’ juxtaglomerular cells, to form AngI. AngI is then hydrolyzed by ACE, predominantly expressed by endothelial cells in the vascular territory of the lungs, to form the biologically active AngII. In addition to AngII, other truncated bioactive peptides have been identified, such as AngIII, AngIV, Ang(1–7), Ang(1–9), AngA, and alamandine. AngII interacts with two seven-transmembrane receptors, AT1R and AT2R, both of which also mediate the effects of AngA. Ang(1–7) mainly acts via the MAS receptor (MASR), and alamandine binds and signals through MRGD (MAS-related G protein– coupled receptor D). IRAP (insulin-regulated membrane aminopeptidase; also known as AT4R) is a binding site for AngIV (*1*–*7*). APA, aminopeptidase A; APN, aminopeptidase N; DC, decarboxylase; MLDAD, mononuclear leukocyte-derived aspartate DC; NEP, neutral endopeptidase; PEP, prolyendopeptidase.

The circulating renin-angiotensin system (RAS) is mainly known for its pivotal role in maintaining cardiovascular homeostasis and fluid and electrolyte balance. In addition, a local RAS is expressed in many tissues and mainly acts at the cellular level, where it mediates cell proliferation, growth, and metabolism. The local RAS works synergistically and independently of the systemic RAS. Angiotensin II (AngII) is the main effector and maintains tissue homeostasis by exerting regulatory and counterregulatory effects through its different receptors. Alternative peptide-receptor axes also assist in maintaining this balance (*1*–*7*). Figure 1 provides an overview of the main components of the RAS. Dysregulation of the RAS, for example, by overexpression of certain RAS components [such as renin, Ang-converting enzyme (ACE), or AngII type 1 receptor (AT1R)], can be involved in the pathophysiology and progression of a broad range of diseases, such as arterial hypertension, kidney disease, and other cardiovascular conditions (*5*, *8*, *9*).

The discoveries of captopril—the first orally active ACE inhibitor (ACEi)—in the mid-1970s (*10*) and losartan—the first orally active, selective AT1R blocker (ARB)—around a decade later (*11*) represent milestones in the history of the RAS. Numerous ACEis and ARBs have been developed since then. Now, ACEis and ARBs are the most common inhibitors of the RAS and are widely used in the management of several diseases, such as arterial hypertension, heart failure, myocardial infarction, and chronic kidney disease (*12*–*15*). Direct renin inhibitors (such as aliskiren) represent a third class of RAS-acting agents and have been added to the armamentarium more recently (*16*). A list of RAS inhibitors (RASi) approved by the U.S. Food and Drug Administration (FDA) is provided in table S1.

After being in clinical use for more than two decades in nonmalignant diseases, ACEi/ARBs have recently received considerable attention in oncology. A large-scale meta-analysis (*17*), published in 2010, found an increased overall occurrence of cancer in ARB users. However, two other meta-analyses published subsequently did not confirm these data (*18*, *19*). The FDA also rebutted these findings with their own meta-analysis (*20*) and an integrated analysis of all 19 rodent carcinogenicity assays of ARBs (*21*). Thus, the data to date do not support an association between ACEi/ARB use and an increased cancer risk. However, they do not suggest a reduced occurrence of cancer either.

Of interest, an increasing number of preclinical studies support the involvement of RAS signaling in cancer development, growth, and progression (*4*). These data have led to investigations of the effects of RASi— both retrospectively and prospectively—in patients with different types of cancer. Interim analysis of a recent phase 2 trial—stemming from our preclinical findings (*22*)—showed encouraging R0 (microscopically margin-negative) resection rates in patients with locally advanced pancreatic ductal adenocarcinoma (PDAC) receiving neoadjuvant losartan plus chemoradiation (*23*). Moreover, our recent retrospective analysis indicated that RASi use is associated with improved survival of patients with nonmetastatic PDAC, presumably by stimulating the tumor’s immune microenvironment, normalizing its extracellular matrix (ECM), and reducing the malignant potential of cancer cells (*24*).

In light of these emerging data, we discuss the role of the RAS in cancer biology with a special emphasis on tumor immunity. In addition, by carefully analyzing the studies with positive versus negative outcomes, we make a case for targeting the RAS to improve treatment of certain malignancies. Moreover, RASi may not only improve the outcome of immunotherapies but also reduce or even prevent adverse effects associated with these therapies.

### The AngII/AT1R axis shapes the tumor microenvironment and promotes an immunosuppressive milieu

Components of the RAS are expressed in various human cancers and cell lines (*4*). Overexpression of AT1R is typically associated with more aggressive tumor features (larger tumors, higher grade, and higher vascular density) and worse outcomes (*25*–*29*).

Moreover, RAS components are also expressed in many cell types of the tumor microenvironment, such as endothelial cells, fibroblasts, monocytes, macrophages, neutrophils, dendritic cells, and T cells (*4*, *30*–*34*). RAS signaling in these cells can facilitate or hinder growth and dissemination and has been shown to affect cell proliferation, migration, invasion, metastasis, apoptosis, angiogenesis, cancer-associated inflammation, immunomodulation, and tumor fibrosis/desmoplasia (*1*, *4*). Generally, the AngII/AT1R axis is considered to favor tumor growth, whereas AngII/AT2R and Ang(1–7)/MAS signaling have opposing effects (*1*, *4*). However, there are also conflicting reports suggesting potential tumor type–specific differences (*35*–*39*).

The tumor-promoting actions of the ACE/AngII/AT1R axis, the main target of classical RASi, have been reviewed elsewhere (*1*, *4*). In this section, we focus on its role in tumor immunity and propose RASi as an adjunct for immunotherapy. Immune checkpoint inhibitors have recently achieved compelling success in melanoma and other solid tumors (*40*). However, their efficacy is diminished by a major barrier—the immunosuppressive tumor microenvironment (*41*). Here, we review how AngII/AT1R signaling shapes the tumor immune microenvironment by modulating desmoplasia, vasculature, inflammation, and immune cells. We also discuss how RASi could alleviate immunosuppression and enhance the outcome of immunotherapy.

### Tumor desmoplasia and solid stress

**Fig. 2:**
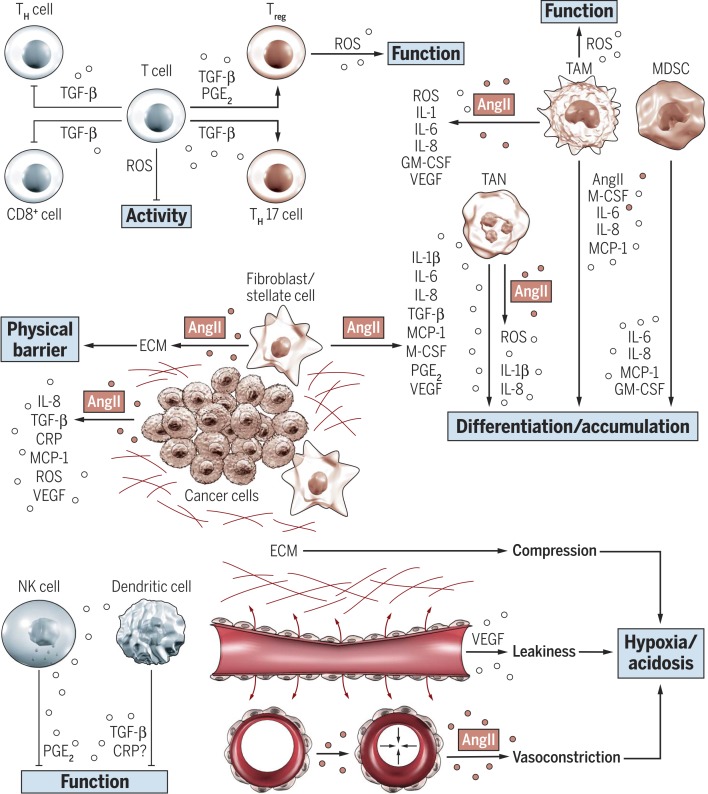
**The AngII/AT1R axis regulates the tumor stroma and contributes to an immunosuppressive microenvironment**. AngII/AT1R signaling can increase production and release of several proinflammatory cytokines in both tumor and stromal cells. Immunomodulatory cytokines regulate a myriad of immunosuppressive immune responses by modulating differentiation, recruitment, and function of both myeloid and lymphoid immune cell types (*4*, *43*, *44*). More precisely, these cytokines suppress the differentiation and function of immunostimulatory cell types [for example, T_H_ (T helper) and CD8^+^ cells, NK cells, and dendritic cells] and activate recruitment and function of tumor-promoting cell types [such as T_regs_, T_H_17 cells, TANs, TAMs (tumor-associated macrophages), and MDSCs (myeloid-derived suppressor cells)]. Fibroblasts are a major source of cytokines and also play a key role in establishing a desmoplastic stroma by production and deposition of ECM. The dense tumor fibrosis represents a physical barrier to immune cell infiltration (*45*) and compresses blood vessels by increasing tissue stiffness and solid stress. The reduced tumor perfusion results in a hypoxic and acidic milieu, which further promotes immunosuppression (*46*–*48*). Vascular endothelial growth factor (VEGF)–induced vascular leakiness (*48*) and AngII-mediated vasoconstriction (*76*, *77*, *80*) further impair tumor perfusion and aggravate hypoxia. GM-CSF, granulocyte-macrophage colony-stimulating factor. PGE_2_, prostaglandin E_2_.

**Fig. 3:**
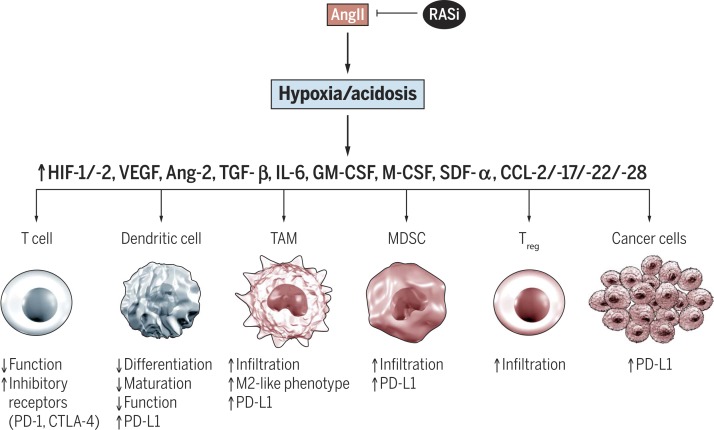
**Tumor hypoxia and acidosis promote immunosuppression**. AngII/AT1R-mediated effects on tumor vasculature (shown in Fig. 2) can impair tumor perfusion and oxygenation, resulting in hypoxia and acidosis within the tumor stroma. The resulting up-regulation of various cytokines, growth factors, and transcription factors [including HIF (hypoxia-inducible factor), VEGF, and TGF-b] enhances an immunosuppressive microenvironment, characterized by impaired T and dendritic cell function, accumulation of immunosuppressive cell types (M2-like macrophages, MDSCs, and T_regs_), and increased expression of inhibitory immune checkpoint molecules such as PD-L1 in tumor and immune cell types (*48*–*50*, *68*). Ang-2, angiopoietin-2; CCL, CC chemokine ligand; CTLA-4, cytotoxic T lymphocyte– associated protein 4; SDF, stromal cell–derived factor.

By regulating cancer-associated fibroblasts (CAFs) and profibrogenic pathways [such as transforming growth factor–β (TGF-β)], the RAS plays a key role in establishing a desmoplastic environment (*22*, *42*), which affects the immune response in multiple ways (Fig. 2). CAFs can manipulate the immune system directly by inhibiting T and NK (natural killer) cell functions, promoting accumulation of suppressive cell types, and maintaining an inflammatory protumorigenic milieu (*43*). TGF-β can also directly induce immune suppression by inhibiting the T cell response (*44*). Dense tumor fibrosis represents a physical barrier to T cell infiltration (*45*). It also compresses blood vessels by increasing solid stress (*46*, *47*). The reduced tumor perfusion results in a hypoxic and acidic milieu, which promotes reprogramming of macrophages into an immunosuppressive phenotype, impairs tumor killing functions of immune cells, and up-regulates the expression of inhibitory immune checkpoint molecules, such as programmed death-ligand 1 (PD-L1), by immune, stromal, and tumor cells (Fig. 3) (*46*–*51*). Normalizing the desmoplastic milieu (for example, by targeting profibrotic pathways and CAFs) can improve the efficacy of immunotherapy (*52*–*54*).

Several studies have demonstrated that RASi can successfully normalize the fibrotic stroma. Co-injection of cancer cells with stromal cells increases tumor size and fibrosis, and treatment with ARBs attenuates these effects (*55*, *56*). Losartan inhibits collagen I production by CAFs and reduces stromal collagen and hyaluronic acid (HA) in several desmoplastic tumor models by decreasing profibrotic signaling via TGF-β, connective tissue growth factor, HA synthase 1 and 3, and endothelin-1 (*22*). Therefore, losartan reduces solid stress and improves vascular perfusion, resulting in decreased tumor hypoxia and improved distribution and efficacy of anticancer drugs and nanotherapeutics (*22*, *42*). Similarly, inhalation delivery of losartan and telmisartan reduces active TGF-β and collagen I expression and increases the intratumoral distribution of nanoparticles (*57*, *58*). Moreover, the cross-talk between tumor-associated neutrophils (TANs), adipocytes, and pancreatic stellate cells (PSCs) promotes tumor desmoplasia and pancreatic cancer growth in obesity (*59*). AT1R inhibition attenuates obesity-induced fibrosis and tumor progression and improves response to chemotherapy (CHT). The AT1R blockade also reduces TANs and regulatory T cells (T_regs_) and increases CD8^+^ T cells through inhibition of PSC activation and subsequent reduction of interleukin-1β (IL-1β) expression (*59*). In another orthotopic model of pancreatic cancer, inhibition of aberrant TGF-β activity by losartan reduced collagen deposition and accumulation of T_regs_ (*60*).

Collectively, these data support the idea that targeting AngII/AT1R signaling with RASi can effectively reduce tumor desmoplasia and thereby decrease solid stress, increase tumor perfusion, reduce hypoxia, enhance T cell infiltration and antitumor immunity, and improve delivery and efficacy of anticancer drugs. Thus, inhibiting the AngII/AT1R axis appears to be an attractive strategy, especially for highly desmoplastic tumors, such as PDAC and some subtypes of breast and lung cancer, and RASi may represent a promising combination partner for immunotherapy.

### Angiogenesis and tumor vasculature

Considerable evidence suggests that AngII/AT1R signaling promotes VEGF-mediated angiogenesis in solid tumors. AT1R expression correlates with VEGF and VEGF receptor (VEGFR) expression and microvessel density (MVD) in different human tumors (*26*, *27*, *29*). In experimental studies, AngII promoted VEGF expression in tumor (*61*–*63*) and stromal cells (*64*). Treatment with either ACEi or ARB reduced VEGF expression and decreased MVD and neovascularization in vivo (*65*, *66*). VEGF also induces vascular hyperpermeability, one of the main characteristics of the abnormal tumor vasculature (*46*, *48*). Tumor vessel leakiness promotes tumor hypoxia and acidosis by impairing tumor blood flow (Fig. 2) (*48*, *67*). As mentioned above, hypoxia helps to create an immunosuppressive milieu (Fig. 3) and promotes tumor progression and dissemination (*48*, *68*). Tumor vessel normalization can alleviate hypoxia, reprogram the immunosuppressive microenvironment, and improve the efficacy of immunotherapy in mice (*68*, *69*). Glioblastoma patients who show enhanced tumor blood perfusion under anti-angiogenic therapy have markedly prolonged survival compared to subjects who experience no change or a decrease in perfusion (*70*–*72*). RASi also reduces VEGF-mediated vascular leakiness in the dermis and retina of rodents (*73*, *74*).

In an orthotopic model of PDAC, inhibition of aberrant TGF-β signaling by losartan restored vessel diameter and permeability (*60*). In a retrospective study of glioblastoma patients receiving anticancer therapy, a concomitant treatment with matrix-depleting antihypertensive drugs improved vascular function as assessed by magnetic resonance imaging (*75*). The impaired perfusion and hypoxic condition of tumors can be further aggravated by AngII-induced vasoconstriction and increased vascular resistance (Fig. 2) (*76*, *77*). Our laboratory has shown that AngII transiently enhanced tumor blood flow and interstitial fluid pressure by increasing the mean arterial blood pressure in different tumor types (*78*, *79*). However, Thews and colleagues (*80*) found that AngII infusion decreased tumor perfusion and oxygenation in small subcutaneous sarcomas but increased both parameters in large tumors. They concluded that perfusion decreased due to vasoconstriction of preexisting functionally intact host vessels in small sarcomas, whereas the newly formed tumor vessels in large tumors did not seem to have this vasoresponsive capability, possibly due to lack of smooth muscle cells and/or angiotensin (AT) receptors (*80*).

Together, available data indicate that AngII/AT1R signaling impairs tumor blood supply through multiple mechanisms, such as desmoplasia-mediated vessel compression, VEGF-induced vessel leakiness and abnormal morphology, and AngIImediated vasoconstriction of host vessels. The resulting tumor hypoxia aggravates immunosuppression and evasion. Although RASi can reduce VEGF-mediated angiogenesis and desmoplasia, additional studies are needed to ascertain whether RASis have the ability to normalize the tumor vasculature, similar to anti-VEGF agents (*48*).

### Inflammation and immune cell modulation

The RAS promotes cancer-related inflammation and infiltration of tumor-promoting immune cells (*1*, *4*, *81*), both of which enhance the immunosuppressive microenvironment (*41*, *82*). Here, we discuss how the RAS modulates the expression of inflammatory cytokines and orchestrates the recruitment of cancer-associated immune cells to the tumor microenvironment.

#### Inflammatory cytokines

A number of studies have shown that AngII/AT1R signaling can increase the production and release of several proin-flammatory cytokines in both tumor and stromal cells (*4*). Fibroblasts represent a main target of the RAS and play a pivotal role in maintaining an inflammatory response. Cytokines released from tumor and stromal cells upon AT1R activation by AngII include TGF-β, IL-1a, IL-1β, IL-6, IL-8, MCP-1 (monocyte chemoattractant protein–1), M-CSF, COX-2 (cyclooxygenase-2), and CRP (C-reactive protein) (Fig. 2) (*4*, *22*, *42*, *56*, *59*, *65*, *83*–*87*). Immunomodulatory cytokines (such as TGF-β, IL-1β, MCP-1, IL-6, and IL-8) can up-regulate multiple—mostly immunosuppressive— pathways by modulating the differentiation and recruitment of both myeloid and lymphoid immune cell types (Fig. 2) (*44*, *82*, *88*–*91*). COX-2 suppresses antitumor immunity and contributes to resistance to immunotherapy, mainly through prostaglandin E_2_ synthesis (*92*, *93*). The role of tumor-derived CRP in tumor immunity is less clear, but it may impair dendritic cell function by reducing their migration activity (*94*).

Oxidative stress represents another aspect of cancer-related inflammation. Although reactive oxygen species (ROS) are involved in T cell activation (*95*, *96*), exposure to ROS can reduce T cell fitness (*90*, *97*, *98*) and enhance the function of T_regs_ (99) andTAMs (*100*). TAMs typically show a polarized M2-like phenotype and contribute to immunosuppression, whereas M1-like macrophages are known to induce antitumor immunity (*101*). AngII/AT1R signaling induces ROS generation in tumor cells and stromal cells (*4*). In prostate cancer cells, AngIImediated expression of oxidative stress–related proteins (such as inducible nitric oxide synthase) and the generation of the ROS family member O_2_^−^ radical are attenuated by the ARB candesartan (*102*).

#### Immune cells

Several studies have shown that RASi can reduce infiltration of TAMs. In human prostate cancer, high MCP-1 and macrophage infiltration are associated with more aggressive tumor features, and MCP-1 independently correlates with prostate-specific antigen recurrence (*103*). AngII/AT1R signaling promotes production and infiltration of TAMs in experimental tumor models; inhibition of AngII production or AT1R signaling down-regulates MCP-1, restrains tumor-induced TAM response, reduces tumor growth, and prolongs survival (*34*, *103*–*105*).

AngII/AT1R signaling is also important for myeloid differentiation and functional maturation (*106*). ACE knockout mice show enhanced extramedullary myelopoiesis and increased numbers of cells with MDSC phenotype (*32*). In contrast, cultured bone marrow from ACE 10/10 mice, a mouse line overexpressing ACE in monocytic cells, demonstrates enhanced myeloid maturation and reduced MDSC production; macrophages from these mice have a more proinflammatory phenotype and more antitumor activity compared to those from wild-type mice (*107*). Similarly, tumor-bearing ACE 10/10 mice showed enhanced immune response, which ultimately resulted in a reduced tumor growth. Notably, ACEi reversed the beneficial effects on tumor growth, but AT1R blockade did not, suggesting that the effects of ACE overexpression were not dependent on AngII/AT1R signaling (*108*, *109*).

Together, available data clearly demonstrate that AngII/AT1R signaling stimulates the expression of different cytokines and growth factors from tumor and stromal cells, which enhance cancer-related inflammation and promote an immuno-suppressive microenvironment (Fig. 2). Beyond the tumor immune microenvironment, the AngII/AT1R axis is also crucial for the maturation and function of immunostimulatory myeloid cells, and ACE overexpression in monocytic cells enhances antitumor immunity, although the latter effect seems to be independent of the AngII/AT1R axis. These conflicting data highlight the complexity of the RAS in cancer immunity. However, because studies supporting a stimulatory role of RAS in tumor immunosuppression considerably outweigh opposing data, we propose that RASi can effectively reprogram the tumor microenvironment toward an immunostimulatory milieu and enhance the efficacy of immunotherapy.

### RASi to reduce side effects of immunotherapy

As discussed above, RASi may increase the intratumoral delivery of T cells and immunotherapeutic agents by modulating tumor vasculature and desmoplasia. This may allow for reduction in the dose of immunotherapeutic agents without decreasing the therapeutic benefit and could ultimately result in a decreased number of severe (grades 3 and 4) immunotherapy-related adverse effects. These side effects can occur in more than 50% of patients, especially if certain checkpoint blockers are combined, and some can be even lifethreatening (*110*, *111*).

Obesity and associated chronic inflammation seem to play a critical role in inducing immunotherapy-associated toxicities (*112*, *113*). Systemic stimulatory immunotherapy, such as αCD40/IL-2, can cause a cytokine storm, characterized by high tumor necrosis factor–α (TNF-α) and IL-6, resulting in multiorgan pathologies and lethality in obese but not in lean mice (*112*, *113*). The TNF blockade ameliorates the observed toxicities in obese mice (*113*). Inhibition of the RAS can also ameliorate chronic inflammation, as shown by reduced serum concentrations of proinflammatory cytokines (TNF-α and IL-6) in patients with hypertension and diabetes (*114*–*116*). This represents another way that RASi may help to reduce or even prevent immunotherapy-induced toxicity.

### RAS inhibition can improve treatment of certain tumors

The effect of RASi on the clinical outcome of patients with different tumor types has been extensively studied in recent years. Tables S2 and S3 provide an overview of the published prospective (*117*–*126*) and retrospective studies (*24*, *127*–*175*), respectively. Here, we summarize the main conclusions based on the available data.

### RASi usage in conjunction with CHT

Available clinical data suggest that RASi may potentiate the effect of certain systemic antitumor therapies. The use of RASi was associated with better outcomes in patients with different solid tumors who received platinum-based CHT (*142*, *143*, *149*, *165*, *172*). The gain in overall survival (OS; the length of time from either the date of diagnosis or the start of treatment that patients are still alive) ranged from ~3 months in advanced non–small cell lung cancer (NSCLC) to 5.7 months in advanced gastric cancer and even 11 months in metastatic colorectal cancer (CRC) (*142*, *149*, *165*, *172*). In line with the clinical data, experimental studies showed that platinum-based CHT can increase VEGF production through up-regulation of AT1R expression. This seems to represent a mechanism for platinum resistance that can be successfully targeted by RASi (*176*, *177*).

In addition, concomitant RASi treatment was associated with better survival in patients with metastatic renal cell carcinoma (RCC; gain in OS, 7 to 26 months) (*137*–*140*), metastatic CRC (gain in OS, ~11 months) (*172*), glioblastoma (*175*), and advanced hepatocellular carcinoma (HCC; gain in OS, ~5 months) (*173*) who received VEGF-targeted therapies. Because AngII/AT1R signaling promotes VEGF-mediated angiogenesis (*4*), RASi may potentiate the effect of anti-VEGF therapy. In a mouse model of Ehrlichs’s ascites carcinoma, the ARB olmesartan augmented the antiangiogenic effect of the tyrosine kinase inhibitor (TKI) sorafenib (*178*). RASi may also represent a strategy to inhibit rapid revascularization (*179*, *180*) and regrowth of tumors (*181*, *182*) after cessation of anti-VEGF therapy, which is often necessary due to treatment-related side effects, especially with VEGFR TKIs (*183*, *184*). Notably, arterial hypertension is a common side effect of anti-VEGF therapy and can be associated with better survival outcomes (*185*). VEGF-targeted therapy-induced hypertension is often treated with RASi, which could represent a potential confounder for the reported beneficial survival results associated with RASi use in patients who received anti-VEGF therapies. However, two points suggest otherwise: First, some studies reported the number of patients who received RASi either at baseline or after initiation of anti-VEGF therapy and showed that most of the patients were taking RASi already at baseline (*137*, *139*). Second, McKay and colleagues (*140*) demonstrated that even in the subgroup of patients who developed anti-VEGF therapy-induced hypertension, RASi users had improved survival compared to nonusers.

Finally, two studies suggested a putative clinical benefit of RASi use in patients who received epidermal growth factor receptor (EGFR) TKIs (*128*, *143*). This could be explained by the preclinical finding that AT1R signaling can regulate proliferation and migration of cancer cells through transactivation of the EGFR by metalloproteinase-dependent shedding of EGF ligands (*4*).

### Tumor characteristics as determinants of RASi efficacy

RASi use was associated with better outcomes in multiple studies, whereas no association was found in others. This suggests that response to RASi treatment may also vary by tumor type and depend on certain tumor characteristics, as discussed below.

In breast cancer, only 2 of 13 studies shown in tables S2 and S3 reported beneficial effects of RASi use, whereas 3 studies found worse outcomes. A meta-analysis found no association of ACEi/ARB use with disease-free survival (DFS; the length of time after primary treatment for a cancer ends that the patient survives without any signs or symptoms of that cancer) or OS in breast cancer (*186*). The heterogeneity in terms of tumor stage, hormone receptor status, human epidermal growth factor receptor 2 overexpression, and (neo)adjuvant treatment regimen could have masked a potential benefit of RASi in certain subgroups and highlights the need for careful patient selection to obtain homogenous and comparable study cohorts.

The use of RASi was associated with better outcomes in patients with RCC, CRC, and HCC (tables S2 and S3). These tumors are well known to respond to anti-VEGF therapy (*187*–*189*). As discussed earlier, RASi may enhance the efficacy of VEGF-targeted therapies and thereby improve clinical outcome. However, in HCC (*125*, *126*, *159*, *164*) and some CRC (*167*) and RCC (*144*) studies listed in tables S2 and S3, most of the patients were not treated with anti-VEGF treatment, suggesting that anti-VEGF–responsive tumors generally seem to be more sensitive to RASi.

RASi therapy had a clinical benefit in both slowly progressing cancers, such as prostate cancer, and highly aggressive tumor types, such as glioblastoma and pancreatic cancer (tables S2 and S3). A phase 2 study at the Massachusetts General Hospital (MGH) is currently investigating whether adding losartan to CHT (FOLFIRINOX), followed by chemoradiation, can convert locally advanced PDAC to resectable tumors (*23*). Preliminary results from this trial showed that R0 resection was achieved in 13 of 25 patients (52%), which is a major improvement compared to previously reported R0 resection rates obtained with neoadjuvant FOLFIRINOX and radiation in locally advanced PDAC (23 to 24%) (*190*, *191*). The median OS was 33 months, with a 2-year survival rate of 65% for all patients and 83% for resected patients (*23*).

In addition, RASi use was effective in both early and advanced tumor stages. In some tumor types, the effect of RASi was investigated primarily in either early tumors (such as resected urinary tract cancer) (*130*, *147*, *150*, *151*) or advanced stages (such as metastatic NSCLC) (*142*, *149*). In RCC and CRC, positive outcomes were reported for both early (*144*, *167*) and metastatic diseases (*137*–*140*, *172*). Notably, in PDAC, a survival benefit in RASi users was only shown for locally advanced/metastatic diseases treated with CHT (*168*–*170*) but not for resected early/locally advanced tumors (*174*).

In contrast, in our own retrospective analysis, RASi use was associated with longer OS in pancreatic cancer patients with resected primary tumors (median OS, 36.3 versus 19.3 months) and locally advanced tumors (median OS, 11.3 versus 9.3 months) but not in meta-static patients. To obtain mechanistic insights, we performed RNA sequencing expression profiling of prospectively collected cancer treatment–naïve pancreatic cancer samples (four lisinopril-treated patients versus four controls). Our data suggest that lisinopril, which was the most commonly used ACEi in our cohort, normalized the ECM, down-regulated genes involved in cancer progression (such as Wnt and Notch signaling), and up-regulated genes associated with the activity of T cells and antigen-presenting cells. In addition, we identified a predictive gene signature for RASi-mediated survival, which was validated in two publicly available cohorts (*24*). A recently published meta-analysis pooling data on different solid tumor types (*192*) showed that the use of ACEi or ARB was associated with improved DFS and OS. After pooling studies that were classified as early (I/II) or advanced (III/IV) stage-dominant, the association with DFS remained significant in both stages (*P* = 0.04 and *P* = 0.03, respectively); a positive association with OS was only observed in advanced tumor stage (*192*).

Finally, HCC usually develops in patients with underlying liver fibrosis/cirrhosis (*193*). The peritumoral liver tissue and the severity of liver dysfunction determine prognosis of HCC, and complications of cirrhosis (portal hypertension and variceal bleeding) are a common cause of death in patients with HCC (*193*). The AngII/AT1R axis plays a crucial role in the pathophysiology of liver cirrhosis (*194*), and RASi can improve both liver fibrosis (*195*) and portal hypertension (*196*). These effects, in addition to the direct antitumor effects of RASi, may also contribute to the improved outcome observed in HCC patients treated with RASi (*125*, *126*, *159*, *164*, *173*).

## CONCLUSIONS

Preclinical studies have provided compelling evidence that the AngII/ AT1R axis regulates almost all hallmarks of cancer. RASi can directly attenuate tumor growth and dissemination and improve the efficacy of systemic therapies by increasing drug delivery to the tumor tissue. The latter should help to reduce the dose of CHT and immunotherapy without decreasing the benefit and consequently decrease the anticancer therapy–induced side effects.

It is also clear that AngII/AT1R signaling contributes to the immuno-suppressive tumor microenvironment in multiple ways. The immuno-suppressive milieu is a major barrier for immunotherapy and may explain why immune checkpoint inhibitors have failed in some tumor types, such as PDAC, and have benefited only a fraction of patients in other indications where these agents are approved. Studies have shown that AT1R inhibition can decrease infiltration of immunosuppressive cell types and increase the number of effector T cells. This could also help to reduce the dose of immunotherapy without lowering drug efficacy, eventually resulting in a decreased number of severe immunotherapy-induced side effects. Although not yet studied in the context of tumor immunity, the AngII/AT1R axis is also important for the maturation of immune effector cells.

Multiple clinical studies have also revealed that RASi may have beneficial effects in a broad range of malignancies. The gain in survival is tumor type– and stage-dependent and ranged from 3 months (advanced NSCLC) to more than 25 months (metastatic RCC) in retrospective studies. However, response to RASi treatment may not only vary with tumor types but also depend on certain tumor characteristics, cancer treatment, and RASi type and dosing. More precisely, RCC, HCC, PDAC, glioblastoma, urinary tract cancer, and NSCLC seem to belong to the responsive tumor types, whereas breast cancer is rather unresponsive to RASi. With respect to cancer treatment, RASi use was associated with better outcomes in patients with NSCLC, gastric cancer, and CRC who received platinum-based CHT and in those with RCC, HCC, and CRC treated with anti-VEGF therapy (for example, sunitinib). More data are needed for other tumor types, such as melanoma, thyroid cancer, head and neck cancer, and hematologic malignancies.

Because the clinical evidence largely came from retrospective studies and small prospective pilot trials, these findings should be considered as hypothesis-generating. However, given the large amount of preclinical and clinical data suggesting a beneficial effect of RASi in different cancer types, we propose that RASis have a great potential to become an adjunct within the oncological armamentarium. Ongoing trials testing whether RASi can improve the antitumor effect of certain anticancer treatments are listed in table S4.

### Future perspectives and translational challenges

Advancing the promising strategy to reprogram the tumor microenvironment with RASi to enhance anticancer treatment will require a close interplay between basic and clinical research and addressing a number of outstanding questions. Preclinical research should combine immune checkpoint inhibitors or other immunotherapy approaches with RASi to confirm whether RASis have the potential to reprogram the immunosuppressive microenvironment and eventually render tumors more sensitive to immunotherapies. In addition, mechanistic studies should not only focus on effects of RASi on the tumor stroma but also investigate treatment-related changes within immune cell populations in the bone marrow and lymphoid organs. This will help to better understand the role of the RAS in cancer immunity.

Moreover, clinical pilot studies focusing on biological readouts— such as intratumoral ECM deposition, immune cell infiltration, and drug distribution—should be designed to confirm the available preclinical data and to pave the way for large randomized controlled efficacy trials. These studies should seek to identify those patients who may benefit most from concomitant RASi use. Such personalized approaches require a tight integration between measurements of various biomarkers —circulating (profibrotic molecules, immune cells, and chemokines), tissue (profibrotic molecules, collagen, and HA), and imaging (perfusion, oxygenation, and drug distribution)—and the treatment outcome (*197*). Assessing the intratumoral expression of the components of the RAS may also have the potential to predict response to RASi treatment.

Finally, the beneficial response of tumors to RASi is dose-dependent. For example, the collagen content of desmoplastic tumors decreases with an increasing dose of ARBs (*42*). However, increasing the dose can cause hypotension and other adverse effects. One potential solution to this challenge is to develop nanoformulations of RASi that will preferentially deliver RASi to the tumor microenvironment. Addressing these issues and challenges will unravel the complexity of RAS signaling and its role in different malignancies and enable development of new strategies to deliver RASi to tumors in safe doses with an even better outcome.

## Supplementary Material

Targeting the renin-angiotensin system to improve cancer treatment: Implications for immunotherapy

## SUPPLEMENTARY MATERIALS

www.sciencetranslationalmedicine.org/cgi/content/full/9/410/eaan5616/DC1

Table S1. RASi approved by the FDA.

Table S2. Published prospective studies using RASi in different types of cancer.

Table S3. Published retrospective studies using RASi in different types of cancer.

Table S4. Ongoing prospective studies investigating the effect of RASi in solid malignant tumors.
