# Supplementary material for: Targeting the renin-angiotensin system to improve cancer treatment: Implications for immunotherapy
Source: Sci Transl Med. 2017 Oct 4;9(410):eaan5616. doi: 10.1126/scitranslmed.aan5616 (PMC5928511; doi:10.1126/scitranslmed.aan5616)
Supplement: Targeting the renin-angiotensin system to improve cancer treatment: Implications for immunotherapy [file STM-09-eaan5616-s001.pdf]

Science Translational Medicine
AAAS

www.sciencetranslationalmedicine.org/cgi/content/full/9/410/eaan5616/DC1

Supplementary Materials for

Targeting the renin-angiotensin system to improve cancer treatment: Implications for immunotherapy

Matthias Pinter and Rakesh K. Jain*

*Corresponding author. Email: jain@steele.mgh.harvard.edu

Published 4 October 2017, *Sci. Transl. Med.* **9**, eaan5616 (2017)
DOI: 10.1126/scitranslmed.aan5616

This PDF file includes:

Table S1. RASi approved by the FDA.
Table S2. Published prospective studies using RASi in different types of cancer.
Table S3. Published retrospective studies using RASi in different types of cancer.
Table S4. Ongoing prospective studies investigating the effect of RASi in solid malignant tumors.

SUPPLEMENTARY MATERIALS

Table S1. RASi approved by the FDA.

Generic name (year of approval)		
**ACEi**	**ARB**	**DRI**
Captopril (1981)	Losartan (1995)	Aliskiren (2007)
Enalapril (1985)	Valsartan (1996)	
Lisinopril (1987)	Irbesartan (1997)	
Ramipril (1991)	Eprosartan (1997)	
Fosinopril (1991)	Candesartan (1998)	
Benazepril (1991)	Telmisartan (1998)	
Quinapril (1991)	Olmesartan (2002)	
Perindopril (1993)	Azilsartan (2011)	
Spirapril (1994)		
Moexipril (1995)		
Trandolapril (1996)		

A search was performed on January 1, 2017 via the following link:

http://www.accessdata.fda.gov/scripts/cder/drugsatfda/index.cfm

Abbreviations: ACEi, angiotensin-converting enzyme inhibitor; ARB, angiotensin receptor blocker; DRI, direct renin inhibitor; U. S. FDA, Unites States Food and Drug Administration.

Table S2. Published prospective studies using RASi in different types of cancer.

Author (year), PMID, (*Reference*)	Study population	Design	Treatment groups (n)	Main outcomes
	**Prostate cancer**			
Uemura (2005), 16369744, (*124*)	Hormone-refractory	Single-center, nonrandomized	Candesartan and androgen ablation (23)	PSA response/stabilization, n=6/2; median time to PSA progression in responders: 5.5 months; PS improvement, n=5; AT1R expression higher in well-differentiated adenocarcinoma
Ronquist (2009), 18932051, (*121*)	Radical prostatectomy	Single-center, randomized	Captopril (32)<br>Control (30)	PSA recurrence: n=3/32 vs. 10/30; median time to PSA recurrence: 16 vs. 10 months; p=0.034
	**Hepatocellular carinoma**			
Yoshiji (2009), 19501932, (*126*)	Curatively treated	Single-center, randomized	Control (25)<br>VK (18)<br>Perindopril (19)<br>Peridopril+VK (25)	Cumulative recurrence rate significantly reduced for perindopril+VK compared to control; perindopril+VK significantly reduced VEGF and AFP-L3 serum levels
Yoshiji (2011), 21874260, (*125*)	Curatively treated	Single-center, randomized	Control (26)<br>BCAA (16)<br>Perindopril (19)<br>Perindopril+BCAA (28)	Cumulative recurrence rate significantly reduced for perindopril+BCAA compared to control; perindopril+BCAA significantly decreased serum VEGF and sVEGFR2 levels and improved IR
	**Pancreatic ductal adenocarcinoma**			
Nakai (2012), 22515232, (*119*)	Advanced	Single-center,	Gemcitabine+candesartan	ORR and DCR: 0% and

		nonrandomized dose-escalating phase I	(14)	79%; median PFS and OS: 7.6 and 22.9 months
Nakai (2013), 23690239, (*120*)	Advanced	Multicenter, nonrandomized phase II	Gemcitabine+candesartan (35)	ORR and DCR: 11% and 63%; median PFS and OS: 4.3 and 9.1 months; median PFS of candesartan 8mg vs. 16mg: 3.5 vs. 4.6 months (p=0.031)
	**Renal cell carcinoma**			
Tatokoro (2011), 20973869, (*123*)	Advanced	Single-center, nonrandomized phase II	Combination of INF-α, cimetidine, meloxicam, and candesartan or perindopril (51)	Complete/partial response: n=4/7; median PFS and OS:12 and 30 months
	**Breast cancer**			
Holmes (2013), 23649190, (*117*)	Stage I-III	Population-based cohort	Total cohort (4661) ACEi use (478)	No effect on death from BC and death from any cause
Sørensen (2013), 23650417, (*122*)	Stage I-III, resected	Population-based cohort	Total cohort (18733) ACEi/ARB use (3075/1989)	Null associations between ACEi or ARB use and recurrence
	**Miscellaneous**			
Jones (2004), 15162145, (*118*)	Advanced cancer	Nonrandomized[1]	Combination of marimastat, captopril, and fragmin (50)	1 of 10 patients with RCC had PR and 3 had SD at 16 weeks; significant inhibition of PHA-stimulated TNF-α release from lymphocytes and change of several angiogenic markers

Abbreviations: AFP-L3, lectin-reactive α-fetoprotein; AT1R, angiotensin II type-1 receptor; BC, breast cancer; BCAA, branched-chain amino acids; DCR, disease control rate; HCC, hepatocellular carcinoma; INF-α, interferon-α; IR, insulin resistance; ORR, overall response rate; PFS, progression-free survival; PHA, phytohemagglutinin; PMID, PubMed identifier; PR, partial response; PS, performance status; PSA, prostate-specific antigen; RCC, renal cell carcinoma; SD, stable disease; sVEGFR2, soluble vascular endothelial growth factor receptor-2; TNF, tumor necrosis factor; VEGF, vascular endothelial growth factor; VK, vitamin K.

[1] Not stated if single- or multicenter design

Table S3. Published retrospective studies using RASi in different types of cancer.

Author (year), PMID, (*Reference*)	Study population	Design	Total (n)	RASi type (n)	Main findings for RASi use[1]
	**NSCLC**				
Wilop (2009), 19399518, (*149*)	Stage IIIB-IV, CHT	Single-center	287	ACEi/ARB (52)	Longer OS
Wang (2015), 25450873, (*148*)	Stage IIIA-B, RT±CHT	Single-center	673	ACEi/ARB (76/66)[2]	ACEi associated with shorter LRPFS
Aydiner (2015), 26039117, (*128*)	Metastatic, CHT	Single-center (case-control)	117	ACEi/ARB (16/21)[2]	Univariate: longer OS for ARB and ACEi/ARB combined; multivariate (only shown for ARB): no association
Miao (2016), 26883083, (*143*)	Stage I-IIIA/Stage IIIB-IV NSCLC, surgery+CHT/CHT	Single-center	301	ACEi/ARB (52)	Univariate: longer PFS in all patients and subgroup of stage IIIB-IV patients; multivariate: not shown
Menter (2016), 27637408, (*142*)	Stage IIIB-IV, carboplatin and paclitaxel (CP)± Bevacizumab (CPB)	Population-based (propensity score matched)[3]	CP: 1465[4] CPB: 348	CP: ACEi/ARB (273) CPB: ACEi/ARB (78)	Associated with longer OS only in patients treated with carboplatin+paclitaxel without bevacizumab
	**Breast cancer**				
Melhem-Bertrandt (2011), 21632501, (*141*)	Stage I-III, neoadjuvant CHT	Single-center	1413	ACEi/ARB (140)	No significant effect on RFS and OS
Ganz (2011), 21479924, (*135*)	StageI-IIIA, curative	Population-based cohort	1779	ACEi (137) ACEi+BB (66)	Increased recurrence (ACEi alone); increased all-cause mortality (ACEi+BB) compared to ACEi/BB non-users
Chae (2011), 21936625, (*134*)	Stage II/III, curative	Single-center	703	ACEi/ARB (168)	Reduced recurrence (additive effect if combined with statins)
Sendur (2012), 22326438, (*145*)	Stage I-IV, treatment N/R	Single-center	486	ARB (102)	No significant association with DFS and OS
Botteri (2013), 23912960, (*131*)	Early triple-negative, resected	Single-center	800	ACEi/ARB (109/51)[2]	No effect on incidence of BC-related events (recurrence or death from BC)
Chae (2013), 23983819, (*133*)	Stage I-III, neoadjuvant CHT	Single-center	1449	ACEi/ARB (105/54)[2]	Only ARB use associated with longer RFS (not if ACEi/ARB analyzed combined)
Boudreau (2014), 24557337, (*132*)	Stage I-II, resected	Population-based cohort	4216	ACEi (559)	Increased risk of second primary BC
Babacan (2015), 25778296, (*129*)	Pathologic N3, resected	Single-center	218	ACEi/ARB (31)	No significant effect on DFS and OS
Goldvaser (2016), 27544756, (*136*)	Early ER+/HER2-,	Single-center	671	ACEi/ARB (93/46)[2]	No significant association with BC-specific survival, DFS, and OS
	**Renal cell carcinoma**				
Keizman (2011), 21600760, (*139*)	Metastatic, sunitinib	Single-center	127	ACEi/ARB (44)	Longer PFS
Keizman (2014), 24309979, (*138*)	Metastatic, sunitinib	Multicenter (pooled analysis)	278	ACEi/ARB (106)	Longer PFS
McKay (2015), 25724518, (*140*)	Metastatic, systemic	Pooled analysis of phase II and III studies (n=6/6)	4736	ACEi/ARB (1487)	Longer OS compared to other/no antihypertensive drug; longer PFS compared to other antihypertensive drug; longer OS compared to

					RASi nonusers in anti-VEGF subgroup
Izzedine (2015), 25795198, (*137*)	Metastatic, sunitinib	Single-center	213	ACEi/ARB (105)	Longer OS and longer PFS
Miyajima (2015), 25691280, (*144*)	Nonmetastatic, resected	Single-center	557	ACEi/ARB (104)	No RASi use associated with shorter metastasis-free and disease-specific survival
Sorich (2016), 26685869, (*146*)	Metastatic, anti-VEGF/placebo	Pooled analysis of phase III trials (n=2)	1545	ACEi/ARB (385)	Longer PFS (in anti-VEGF-treated subgroup) and OS (in sunitinib-treated subgroup) in univariate but not in multivariate analysis
	**Urinary tract cancer**				
Tanaka (2012), 22187036, (*147*)	Localized UTUC, resected	Multicenter (pooled analysis)	279	ACEi/ARB (48)	No RASi use associated with shorter metastasis-free survival
Yuge (2012), 22872290, (*151*)	NMIBC, resected	Single-center	330	ACEi/ARB (51)	No RASi use associated with increased recurrence
Blute (2015), 26173101, (*130*)	NMIBC, resected	Single-center	340	ACEi/ARB (143)	Longer RFS
Yoshida (2017), 27730369, (*150*)	Bladder cancer, T1-4, N0/≥1, resected	Single-center	269	ACEi/ARB (56)	Longer cancer-specific survival and OS
	**Prostate cancer**				
Alashkham (2016), 27053500, (*127*)	Local/locally advanced, RT+HT	Single-center (propensity score matched)[3]	558	ACEi/ARB (103)	Reduced risk of BR compared to no hypertension-no RASi and hypertension-no RASi groups
	**Pancreatic ductal adenocarcinoma**				
Nakai (2010), 20978506, (*168*)	Advanced, CHT	Single-center	155	ACEi/ARB (27)	Longer OS and longer PFS compared to patients without hypertension
Nakai (2013), 23000889, (*169*)	Advanced, CHT	Single-center	250	ACEi/ARB (69)	Longer OS
Nakai (2015), 25398651, (*170*)	Advanced, CHT	Single-center	349	ACEi/ARB (108)	RASi associated with longer PFS and OS only in never smokers and patients receiving gemcitabine monotherapy (univariate)
Tingle (2015), 26600982, (*174*)	T1-4/N0-1, resected	Single-center	164	ACEi/ARB (41)	No association with OS
Liu (2017), 28600474, (*24*)	Resected, locally advanced, metastatic	Single-center	794	ACEi/ARB (297)	Longer OS in resected and locally advanced pancreatic cancer
	**Esophageal cancer**				
He (2015), 25777421, (*160*)	Stage I-IV, CRT+/-resection	Single-center	1174	ACEi/ARB (350)	No association with OS, EC-specific survival, and non-EC-specific survival
Chen (2015), 24961505, (*156*)	Stage I-III, resected	Single-center	141	ACEi/ARB (20)	Longer OS
	**Gastric cancer**				
Kim (2012), 23052034, (*165*)	Advanced, CHT	Single-center	63	ACEi/ARB (30)	Longer OS
	**Colorectal cancer**				
Heinzerling (2007), 17375777, (*161*)	CRC stage II, surgery	Single-center	55	ACEi (N/R)	Hypertension (but not ACEi use) inversely predicted distant metastasis
Engineer (2013), 24151534, (*158*)	Advanced CRC, CHT/RT	Single-center	262	ACEi/ARB+BB (39)<br>ACEi/ARB (52)	ACEi/ARB+BB use compared to neither ACEi/ARB nor BB use: decreased mortality and

					tumor progression
Osumi (2015), 26807236, (*172*)	Metastatic CRC, CHT	Single-center	181	ARB (104)	Total cohort: longer PFS; patients who received 2nd line CHT: longer OS and PFS
Morris (2016), 27203227, (*167*)	Rectal cancer, neoadjuvant (C)RT	Multicenter (2 sets, separate and pooled analyses)	115<br>186	ACEi/ARB (25)<br>ACEi/ARB (49)	Increased rate of pCR in 2 independent cohorts (univariate), and in combined data set (multivariate)
	**Hepatobiliary cancer**				
Kaibori (2011), 21332549, (*164*)	HCV-related HCC without cirrhosis and with cirrhosis, resected	Single-center	185[5]<br>141	ACEi/ARB (37)<br>ACEi/ARB (20)	No cirrhosis: worse DFS and OS in patients with hypertension without RASi compared to those without hypertension/hypertension plus RASi; cirrhosis: no significant association
Facciorusso (2015), 25974743, (*159*)	HCC, RFA-treated	Single-center	153	ACEi/ARB (49/31)[2]	Only ARB use associated with longer OS and TTR compared to RASi non-use
Nakai (2016), 27630357, (*171*)	Advanced BTC, CHT	Single-center	287	ACEi/ARB (74)	No association with PFS and OS
Pinter (2017), DOI: 10.1177/2050640617695698, (*173*)	HCC, sorafenib/experimental/BSC	Multicenter (2 sets, separate analyses)	156<br>76	ACEi/ARB (43)<br>ACEi/ARB (38)	Longer OS in both independent cohorts
	**Melanoma**				
De Giorgi (2013), 24182700, (*157*)	Nonmetastatic, resected	Single-center	741	ACEi/ARB (126)	No effect on recurrence and death
	**Glioblastoma**				
Carpentier (2012), 22650322, (*154*)	Supratentorial GBM, RT	Single-center	87	ACEi/ARB (18)	Significant steroid-sparing effect at the beginning and during RT
Januel (2015), 26053493, (*163*)	Supratentorial GBM, RT+TMZ	Single-center	81	ACEi/ARB (26)	Longer PFS and OS; steroid-sparing effect of RASi
Kourilsky (2016), 26754004, (*166*)	Supratentorial GBM, newly diagnosed	Single-center	22	ARB (11)	Significantly lower peritumoral T2-FLAIR and ADC compared to matched controls on preoperative MRI (suggesting less edema)
Levin (2017), 28631191, (*175*)	GBM, CHT and/or bevacizumab	Population-based	1186	ACEi/ARB (318)	Longer OS
	**Hematologic malignancies**				
Buchler (2005), 15692600, (*152*)	Multiple myeloma, PBSCT	Single-center	168	ACEi (25)	Total cohort: shorter OS (multivariate); hypertensive patients: shorter OS and PFS (univariate)
Chae (2014), 24628293, (*155*)	AML, CHT	Single-center	1043	ACEi/ARB (88)	No association with OS
	**Several tumor types**				
Holmes (2013), 24075077, (*162*)	Lung<br>CRC<br>Breast<br>Prostate	Population-based	4241<br>3967<br>4019<br>3355	ACEi/ARB (1256)<br>ACEi/ARB (1187)<br>ACEi/ARB (880)<br>ACEi/ARB (956)	Shorter OS for lung cancer and breast cancer compared to antihypertensive drug non-users
Cardwell (2014), 24521426, (*153*)	Breast<br>CRC	Population-based (nested case-	9814[6]<br>4762	ACEi/ARB[7]	ACEi associated with reduced cancer-specific

	Prostate	control)	6339		mortality only in CRC (if fully adjusted)

Abbreviations: ACEi, angiotensin-converting enzyme inhibitor; ADC, Apparent Diffusion Coefficient; AML, acute myeloid leukemia; ARB, angiotensin II receptor type-1 blocker; BB, β-blockers; BC, breast cancer; BR, biochemical recurrence; BTC, biliary tract cancer; CHT, chemotherapy; CRC, colorectal cancer; CRT, chemoradiotherapy; DFS, disease-free survival; EC, esophageal carcinoma; ER, estrogen receptor; GBM, glioblastoma; HCV, hepatitis C virus; HER, human epidermal growth factor receptor; HR, hazard ratio; HT, hormonal therapy; MRI, magnetic resonance imaging; NMIBC, non-muscle-invasive bladder cancer; N/R, not reported; OR, odds ratio; OS, overall survival; PBSCT, peripheral blood stem cell transplantation; pCR, pathologic complete response; PFS, progression-free survival; PMID, PubMed identifier; RFA, radiofrequency ablation; RFS, recurrence-free survival; RR, risk ratio; RT, radiotherapy; FLAIR, Fluid Attenuated Inversion Recovery; TMZ, temozolomide; TTR, time to recurrence; UTUC, upper-tract urothelial carcinoma; VEGF, vascular endothelial growth factor.

[1] Positive/negative associations obtained from multivariate/adjusted analyzes if not indicated otherwise

[2] ACEi and ARB analyzed separately.

[3] A propensity score matched analysis was used to balance covariates.

[4] Two cohorts based on first-line regimen were analyzed separately (carboplatin+paclitaxel without (group 1) and with bevacizumab (group 2))

[5] Total number of study cohort was 469 but ACEi/ARB use was only investigated in 326 patients with HCV (no cirrhosis, n=185; cirrhosis, n=141; analyzed separately)

[6] Nested case-control analysis to compare ACEi/ARB use in cancer patients dying from cancer with up to 5 controls (not dying from cancer); for detailed information please see publication.

[7] ACEi and ARB analyzed separately; number of users depends on type of analysis; for detailed information please see publication.

Table S4. Ongoing prospective studies investigating the effect of RASi in solid malignant tumors.

ClinicalTrials.gov identifier	Condition	Design	Intervention	Primary endpoint	Status
NCT01805453	Newly-diagnosed glioblastoma	RCT, phase III	SOC + Losartan OR placebo	Steroid dose required to control edema	Active, not recruiting
NCT01821729	Locally advanced pancreatic cancer	Single-arm, phase II	FOLFIRINOX + losartan, then proton beam radiation	Proportion free of progression after FOLFIRINOX + losartan	Recruiting
NCT02770378	Recurrent glioblastoma	Single arm, phase I	CUSP9v3 protocol[1]	Dose-limiting toxicity	Recruiting
NCT00086723	Metastatic cancer	Single-arm, phase I/II	rt-PA, captopril	MTD, toxicity, generation of angiostatin	Completed
NCT01276613	Resectable pancreatic cancer	Single-arm	Gemcitabine, losartan	Intratumoral gemcitabine and gemcitabine DNA levels	Active, not recruiting

Abbreviations: MTD, maximum tolerated dose; RAS, renin-angiotensin system; RCT, randomized controlled trial; rt-PA, recombinant tissue plasminogen activator; SOC, standard of care.

[1] CUSP9v3 treatment protocol includes temozolomide, aprepitant, minocycline, disulfiram, celecoxib, sertraline, captopril, itraconazole, ritonavir, auranofin
